# Study of Feasibility and Safety of Higher-Dose Dexmedetomidine in Special Outpatient Examination of Pediatric Ophthalmology

**DOI:** 10.1155/2019/2560453

**Published:** 2019-04-08

**Authors:** Chaoqiao Chen, Minji You, ZhangLiang Li, Li Nie, Yune Zhao, Gang Chen

**Affiliations:** ^1^Sir Run Run Shaw Hospital, School of Medicine, Zhejiang University, Hangzhou 310000, China; ^2^The Eye Hospital of WenZhou Medical University, Wenzhou 325000, China

## Abstract

**Objective:**

To investigate the feasibility and safety of higher-dose dexmedetomidine in ophthalmological outpatient examination of children with cataract.

**Methods:**

100 cases of children were recruited in the study and randomly equally divided into two groups. One group was given 2 *μ*g/kg intranasal dexmedetomidine anesthesia, while the other group was under 3 *μ*g/kg. The dosage of dexmedetomidine was calculated by the same anesthesiologist according to the weight of patient. After sufficient sedation, the same ophthalmologist performed ocular examinations manually, including intraocular pressure, keratometry, axial length, and corneal thickness and recorded the ocular position score during intraocular pressure measurement and corneal thickness measurement. Other variables were sedation onset time, recovery time, vital signs, and side effects.

**Results:**

In intraocular pressure measurement, only one case in the 2 *μ*g/kg group did not complete the examination, while all cases in the 3 *μ*g/kg group completed the examination and the difference of the success rate between the two groups was nonsignificant (*P* > 0.05). The success rates of the 3 *μ*g/kg group in corneal curvature, axial length, and corneal thickness examination were 96%, 92%, and 86%, respectively, which were significantly higher than those of the 2 *μ*g/kg group (22%, 18%, and 4%). The average onset time of sedation in the 3 *μ*g/kg group was 15.42 ± 2.09 minutes, which was significantly shorter than that in the 2 *μ*g/kg group (19.52 ± 2.43 minutes, *P* < 0.001). The average time of completing all examinations in the 3 *μ*g/kg group was 18.36 ± 4.01 minutes, which was significantly shorter than that in the 2 *μ*g/kg group (22.62 ± 4.13 min, *P* < 0.001). The recovery time of group 3 *μ*g/kg was 90.62 ± 27.80 min, which was significantly longer than that of group 2 *μ*g/kg (49.20 ± 15.50 min). Vital signs such as pulse, blood pressure, oxygen saturation, and heart rate kept in normal range throughout the tests, and no obvious side effects were observed.

**Conclusion:**

3 *μ*g/kg intranasal dexmedetomidine had a higher sedation success rate and quality than 2 *μ*g/kg did in pediatric ocular examinations, without any obvious side effects.

## 1. Introduction

Congenital cataract usually requires surgical therapy. Children bearing this disease need special preoperative outpatient examination in order to prepare the intraocular lens, preevaluate the postoperative complications and decide the vision training procedures [1–3]. The basic principle of ophthalmologic examination is to sit-still and fix the ocular bulb. However, the accuracy of the examination can be affected due to the fact that most of the children cannot cooperate well when they feel anxiety, fear, and discomfort. Thus, sedation is an essential part in the examination of children [4]. An international consensus guideline on sedation and analgesia in children recommended the early use of oral chloral hydrate or enteral sedative agent. The clinical application of sedatives has some limitations since sedation depth is often not achieved in great individual variability as well as frequent complications, such as hypoxemia, emesis, and irritation [5].

Dexmedetomidine is a new *α*2-adrenoceptor agonist with sedative, anxiolytic, and analgesic-sparing effects and minimal depression of respiratory function. Dexmedetomidine also exerts its inhibition activity in sympathetic stimulation of children. Compared with other clinical anesthetic drugs, dexmedetomidine has advantages in reducing anesthetics and opioid consumption, postoperative rigors, phrenitis, and dysphoria in analepsia stage [6].

In recent years, intravenous dexmedetomidine has been shown to provide a reliable and effective sedation in clinical trials [7–9]. Gan et al. have reported that 2 *μ*g/kg intranasal dexmedetomidine could rescue sedation after chloral hydrate failure in pediatric ophthalmic examination [10] and the slit-lamp photography [11]. Our previous study has shown that 2 *μ*g/kg intranasal dexmedetomidine can only be used satisfactorily in ophthalmic examination that do not contact cornea, which means that this dosage could not render sufficient sedation depth in higher stimulating examinations requiring cornea contact and eye fixation [10, 11].

In this study, we aim to examine the effect of higher-dose dexmedetomidine (3 *μ*g/kg) in the special outpatient examination of pediatric ophthalmology and evaluate its feasibility and safety.

## 2. Methods and Materials

Children with congenital cataract aged from 6 to 24 months with American Society of Anesthesiologists (ASA) physical status 1-2 were enrolled in this study, either surgery or postoperative examination needed. The physical status of patient was thoroughly evaluated before sedation. Children with congenital heart disease, serious sinus bradycardia, developmental delay, or asthma, pneumonia, respiratory tract infections in latest 3 months, gastroesophageal reflux, facial abnormalities, allergy to dexmedetomidine or chloral hydrate, and weight <2 or >20 kg were excluded.

**Figure 1:**
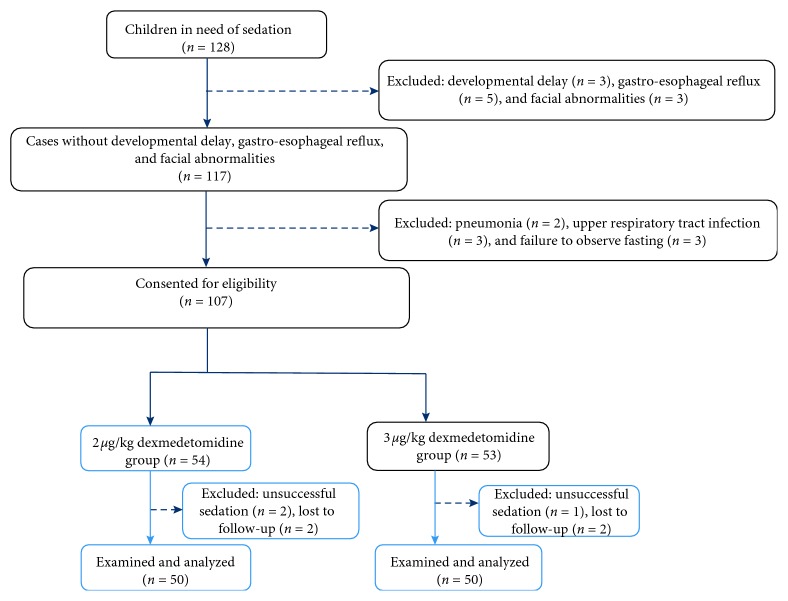
CONSORT diagram showing the flowchart of participants throughout each stage of the randomized trial.

All patients must fast for 8 hr of solid food, 6 hr of rice cereal and formula milk, 4 hr of breast milk, and 2 hr of clear fluids. Dexmedetomidine (Ai Bei Ning; Jiang Su Heng Rui Medicine Co. Ltd, Jiangsu Province, China) was prepared at the commercial concentration of 100 ug/ml and administered in a 1 ml tuberculin syringe by the same doctor. All children were kept in supine position for 1 to 2 min to facilitate nasal absorption. A child not adequately sedated in 30 minutes of dexmedetomidine infusion was considered as a case of unsuccessful sedation; the child was sedated by oral chloral hydrate and excluded from the following examinations. If any unstable vital signs occurred, the patient was quitted from the study and transferred for emergency treatment . A flowchart of this procedure is shown in Figure 1.

**Figure 2:**
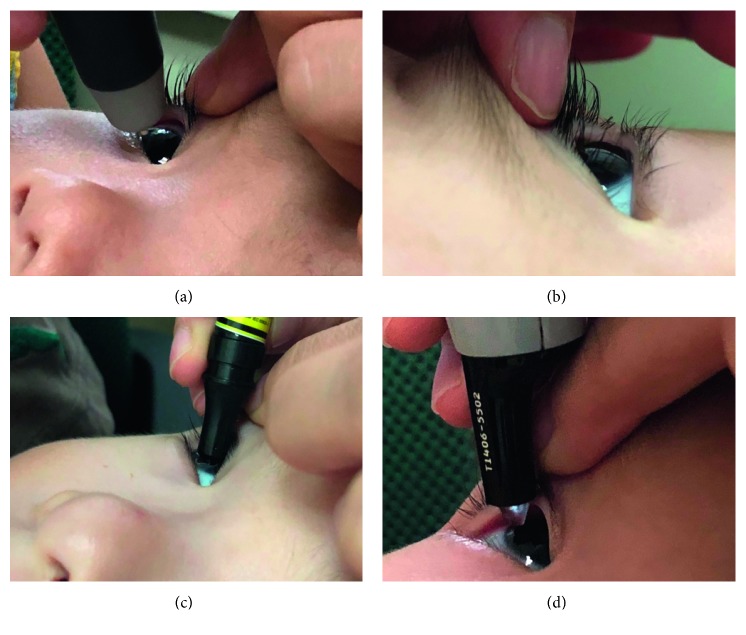
(a) Measurement of IOP: move the tonometer forward slowly and measure the IOP on the center of the cornea 6 times for average. (b) Corneal curvature: the keratometer measures the fully exposed cornea 3 times for average. (c) Axial length (A-scan): measure at the center of cornea 10 times for average. (d) Corneal thickness: choose one of the five positions of cornea (central, temporal, nasal, superior, and inferior) and measure 3 times for average.

After intranasal administrations, one ophthalmologist performed all the tests. The test order was listed as follows: (1) the intraocular pressure (IOP) was measured by a rebound tonometer (SUOER SW-500, Tianjin, China); (2) corneal curvature was measured by a hand-held keratometer (HandyRef-k NIDEK, Japan); (3) axial length was obtained by a contact A-scan (A-scan, Quantel Axis Nano, France); (4) corneal thickness was measured by a hand-held, portable ultrasonic pachymeter (PachPen, Accutome, Inc., Pennsylvania, USA). If one step was failed, then the next one was continued in order. The details of each operation is shown in Figure 2.

**Figure 3:**
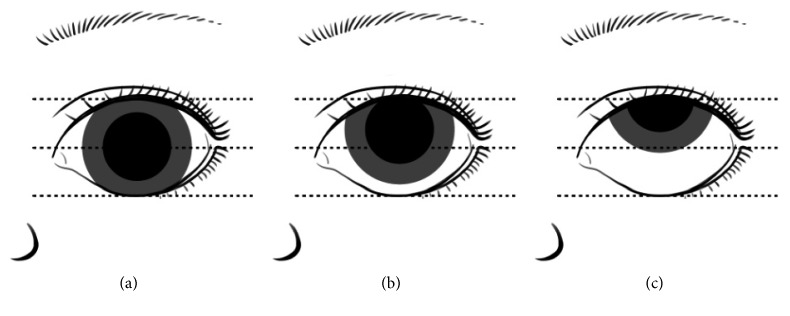
(a) The inner and outer canthus line across the central cornea; (b) inferior limbus does not exceed the inner and outer canthus lines; (c) inferior limbus exceeds the inner and outer canthus line.

The eye position was evaluated during the IOP and corneal thickness measurement by the same ophthalmologist mentioned above [11]. The standard of eye position score is shown in Figure 3.

After intranasal administrations, vital signs including pulse oximetry, blood pressure, oxygen saturation, and heart rate were continuously monitored and recorded every 10 minutes. The exam of presedation blood pressure was omitted due to uncooperative behaviors of the patients. The state of sedation was quantified by a standardized rating scale ranging from MOAAS 6 to 0 (discrete stages of reversible loss of consciousness). MOAAS 4–6 was considered as waking state, while MOAAS 0–3 as sedated enough for tests. The onset time was defined as the duration from intranasal administration to MOAAS less than 3, and the recovery time as from test finished to awaking state. Parents were encouraged to wipe children's face with a chill towel 30 min after completing all examinations. The presence of vomiting, hypoxia, and irritation was surveyed by a nurse via telephone in next 48 hours.

This study was approved by the Ethics Committee of the Eye Hospital, WenZhou Medical University, and registered with the Chinese Clinical Trial registry (http://www.chictr.org.cn/showproj.aspx?proj=24071) (ref: ChiCTR1800014417). Written informed consent was obtained from parents/guardian of eligible children.

### 2.1. Statistical Analysis

In our preliminary study, 20 patients prepared for the corneal curvature test were evenly divided into two groups, rendering 30% and 70% success rates in 2 *μ*g/kg and 3 *μ*g/kg intranasal dexmedetomidine anesthesia, irrespectively. So, patients sample was calculated as 30 in each group through statistic software STATA/SE 12.0 (*α* = 0.05, *β* = 0.1, one-side). We decided 50 cases in each group to assure the validity of this research. Patients were randomly separated into two groups using a computer random number generator with a 1 : 1 allocation (simple randomization, no significant difference, and hidden case), and patient with uneven number was allocated in the 2 *μ*g/kg group, while with even number in the 3 *μ*g/kg group. During this study, quantitative data were expressed as mean and standard deviation, and all categorical data were expressed as frequencies or percentages. Comparisons between quantitative data were made by Wilcoxon two-sample test and categorical data by chi-squared test, through SPSS software for Windows version 22.0 (SPSS Inc., Chicago, IL, USA). A *P* < 0.05 was considered significant difference.

## 3. Results

### 3.1. Demographic Characteristics

A total of 100 patients were enrolled; each group contained 50 patients and the pediatric anesthesia was carried out by the same anesthesiologist (Chen CQ). The demographic characteristics were listed in Table 1. There were no significant differences in age, weight, gender, and ASA physician status between the two groups (*P* > 0.05).

#### 3.1.1. Validity of Examinations

Only one case failed in the 2 *μ*g/kg group, while all cases in the 3 *μ*g/kg group finished the intraocular pressure measurement. The completion rates had no significant difference between the two groups. However, the success rate of corneal curvature, axial length, and corneal thickness tests were 96%, 92%, and 86% in the 3 *μ*g/kg group, respectively, which were significantly higher than those in the 2 *μ*g/kg group (22%, 18%, and 4%, *P* < 0.001, Table 2). The success rate that finished all tests in the 3 *μ*g/kg group was 86%, whereas only 4% in the 2 *μ*g/kg group.

The quality of eye position was evaluated by the same ophthalmologist, which is listed in Table 2. There was a significant difference between the two groups (*P* < 0.001). 44 children in the 3 *μ*g/kg group showed a trend to keep their eye in good position (Score 1-2) without corneal reflex, when compared with only 2 children matching the score in the 2 *μ*g/kg group (*P* < 0.001).

#### 3.1.2. Effectiveness of Sedatives

The MOAAS score of presedation in the 2 *μ*g/kg and 3 *μ*g/kg groups was 2.56 ± 2.11 and 2.65 ± 1.98, respectively, while the recovery score of them was 4.26 ± 1.99 and 4.25 ± 2.08, both of which showed no significant difference (*P* > 0.05). The average sedation onset time in 3 *μ*g/kg was 15.42 ± 2.09 min, which was significantly shorter than 19.52 ± 2.43 min in the 2 *μ*g/kg group (*P* < 0.001). The duration time greatly shortened in the 3 *μ*g/kg group (18.36 ± 4.01 min), compared to the 2 *μ*g/kg group (22.62 ± 4.13 min) (*P* < 0.001). However, the recovery process in the 3 *μ*g/kg group significantly delayed when compared to the 2 *μ*g/kg group (90.62 ± 27.80 min vs 49.20 ± 15.50 min, *P* < 0.001, Table 2).

As shown in Table 3, the heart rate during onset time, duration time, and recovery time in the 3 *μ*g/kg group were significantly lower than those in the 2 *μ*g/kg group. The SpO_2_ in the 3 *μ*g/kg group decreased significantly, compared to the 2 *μ*g/kg group during the examination (*P* < 0.05), but did not differ significantly at other checkpoints. Only one patient showed 94% SpO_2_ but recovered after 10 seconds, who do not require any medical intervention. The blood pressure in presedation time was not recorded because of children's uncooperative behavior. The blood pressure of the 3 *μ*g/kg group was significantly higher than that of the 2 *μ*g/kg group during onset and examined time (*P* < 0.001). However, though many vital signs seemed worse in the 3 *μ*g/kg group than in the 2 *μ*g/kg group, no significant adverse events were observed during the anesthesia, as all of them were in permissible range clinically.

### 3.2. Adverse Events and Safety Analysis

As mentioned above, only one patient in the 3 *μ*g/kg group showed 94% SpO_2_ after 30 min sedation, without further intervention. No hypoxia occurred in all patients, and no poor appetite occurred in 48-hour follow-up. One patient vomited on the way home (in the 3 *μ*g/kg group) but also had a history of car sickness. In addition, no patient showed any irritation or discomfort situation after discharge.

## 4. Discussion

Low-dose dexmedetomidine has been applied to the eye examinations in pediatric outpatient clinic for several years [12]. Congenital cataract usually requires thorough special outpatient examination before or after surgery. Sedation is essential for uncooperative children. Chloral hydrate is easily and wildly used in many countries, whereas the success rate of sedation is low and it should be noted that chloral hydrate often results in many side effects including vomiting and diarrhea [13].

Dexmedetomidine is a highly selective *α*2-adrenergicreceptor agonist with deep sedative effect and shows no respiratory depression [14]. Intranasal bioavailability of dexmedetomidine is 65%, and it is easily and effectively absorbed with no pain or irritation through nasal mucosa [15]. Dexmedetomidine is also widely used during many other clinical practices. Cao et al. has reported that children sedated with intranasal dexmedetomidine displayed higher successful sedation rates and better quality of examinations than with oral chloral hydrate [11].

In this study, the children sedated with 3 *μ*g/kg intranasal dexmedetomidine displayed higher successful sedation rates than those with 2 *μ*g/kg. Similarly, Li et al. have reported that 87% of patients were successfully sedated and finished echocardiography examination under 3 *μ*g/kg intranasal dexmedetomidine [9]. Ibrahim verified that intranasal dexmedetomidine 3 *μ*g/kg could be used safely and efficiently to induce a moderate conscious sedation, facilitating parents' separation and intravenous cannulation successfully [16]. Cao et al. have shown that 61 children sedated with 2 *μ*g/kg intranasal dexmedetomidine rendered a success rate of 85.9% [11]. In our study, both 2 *μ*g/kg and 3 *μ*g/kg groups could successfully complete noncontact ophthalmic examination, but children in the 3 *μ*g/kg group showed particular advantages on corneal curvature, axial length, and corneal thickness examinations.

Dexmedetomidine can not only sedate and ease pain but also decrease the activity of sympathetic nervous system and affect hemodynamics. Low dose of dexmedetomidine can lead to hypotension and bradycardia, while high-dose of dexmedetomidine exhibits severe bradycardia and hypertension. Reynolds et al. showed that when 3 *μ*g/kg intranasal dexmedetomidine in the examination of auditory brainstem responded (ABR), 2 out of 90 cases showed mild decrease of SpO_2_ but recovered rapidly after oxygen inhalation and posture changes [17]. The study of Tug et al. compared two intranasal doses of dexmedetomidine in children for magnetic resonance imaging sedation, namely, 3 *μ*g/kg versus 4 *μ*g/kg. The heart rate and blood oxygen saturation were greatly declined after 30 min of sedatives in both groups, but intragroup comparison showed no significant difference throughout the anesthesia and examination process [18]. In our study, the heart rate and blood pressure obviously decreased in onset and examination time in the 3 *μ*g/kg intranasal dexmedetomidine group, whereas the effectiveness of 3 *μ*g/kg intranasal dexmedetomidine was better than the 2 *μ*g/kg group. All physiological signs of patients were in the normal range according to their ages and need no emergency treatment. Only one patient in the 3 *μ*g/kg group showed 94% of SpO_2_ but recovered after 10 seconds. Furthermore, in 48 hours' follow-up, only one child in the 3 *μ*g/kg group vomited on the way home, most probably due to his car sickness history, while the other patients showed no nausea and vomiting.

However, there are some limitations in our study. First, we only focused on cataract surgery of children, which was only a small part of ophthalmic practice. Second, dexmedetomidine was frequently used in lots of pediatric ophthalmic tests, including preoperation slit-lamp photography, perioperative monitoring, postoperative follow-up examinations, and secondary intraocular lens implantation, so whether patient would show drug resistance in repeated use of intranasal dexmedetomidine needed to be answered.

## 5. Conclusion

Higher dose of intranasal dexmedetomidine (3 *μ*g/kg) showed higher success rate in pediatric ophthalmic examinations, including corneal curvature, axial length, and corneal thickness tests, without any significant side effects, when compared with the dose of 2 *μ*g/kg. It is a feasible and safe method for the ophthalmologist to collect accurate data in outpatient practice.

## Figures and Tables

**Table 1:** Patients' demographics in two groups.

Groups	2 *μ*g/kg (*n*=50)	3 *μ*g/kg (*n*=50)	*P* value
Age (month)	12.3 ± 4.9	12.4 ± 5.0	0.914
Weight (kg)	10.1 ± 1.4	10.2 ± 1.4	0.806
Gender (male : female)	24 : 26	26 : 24	0.689
ASA (Ι : Π)	49 : 1	50 : 0	0.315

**Table 2:** Ocular examination success rate, eye position score, and parameters of sedation time in both groups.

Group	2 *μ*g/kg group (*n*=50)	3 *μ*g/kg group (*n*=50)	*P* value
*Total number of finished patients*			
Intraocular pressure	49 (98%)	50 (100%)	0.315
Corneal curvature	11 (22%)	48 (96%)	<0.001
Optic axis	9 (18%)	46 (92%)	<0.001
Corneal thickness	2 (4%)	43 (86%)	<0.001

*Eye position score of intraocular pressure examination*			
Score 1	10	38	
Score 2	35	10	
Score 3	5	2	<0.001

*Eye position score of corneal thickness measurement*			
Score 1	0	21	
Score 2	4	23	
Score 3	46	6	<0.001

Duration of examination (min)	22.62 ± 4.13	18.36 ± 4.01	<0.001

Onset time (min)	19.52 ± 2.43	15.42 ± 2.09	<0.001

Recovery time (min)	49.20 ± 15.50	90.62 ± 27.80	<0.001

**Table 3:** Vital signs in different stages between 2 *μ*g/kg and 3 *μ*g/kg groups.

Group	2 *μ*g/kg group (*n*=50)	3 *μ*g/kg group (*n*=50)	*P* value
*Heart rate*			
Presedation	123.0 ± 8.8	121.8 ± 9.6	0.545
Onset	110.7 ± 10.0	93.2 ± 7.7	<0.001
Examination	108.4 ± 6.6	96.2 ± 6.7	<0.001
Recovery	121.0 ± 11.0	102.1 ± 8.2	<0.001

*SpO*_*2*_			
Presedation	98.8 ± 0.7	98.8 ± 0.7	0.851
Onset	97.6 ± 0.8	97.3 ± 0.7	0.072
Examination	97.38 ± 1.03	96.92 ± 1.03	0.014
Recovery	98.38 ± 1.05	97.98 ± 1.41	0.230

*Mean arterial pressure*			
Onset	83.22 ± 1.91	80.84 ± 3.15	<0.001
Examination	83.22 ± 1.91	81.70 ± 2.52	0.001
Recovery	83.22 ± 1.91	82.84 ± 1.89	0.308

## Data Availability

The data used to support the findings of this study are included within the article.
